# Involvement of Auxin, Flavonoids and Strigolactones in the Different Rooting Ability of European Chestnut (*Castanea sativa*) and Hybrids (*Castanea crenata* × *Castanea sativa*)

**DOI:** 10.3390/plants13152088

**Published:** 2024-07-27

**Authors:** Petra Kunc, Aljaz Medic, Gregor Osterc

**Affiliations:** Department of Agronomy, Biotechnical Faculty, University of Ljubljana, Jamnikarjeva 101, SI-1000 Ljubljana, Slovenia; petra.kunc@bf.uni-lj.si (P.K.); aljaz.medic@bf.uni-lj.si (A.M.)

**Keywords:** adventitious roots, phenolic compounds, phytohormones, vegetative propagation, woody plants

## Abstract

The aim of this study was to investigate the differences between *Castanea sativa* Mill. and *Castanea crenata* Siebold & Zucc. × *Castanea sativa* Mill. in rooting ability in relation to endogenous levels of auxin, auxin cofactors and inhibitors that influence rooting success. Leafy cuttings of the two commercial cultivars ‘Marsol’ and ‘Maraval’ (*Castanea crenata* × *Castanea sativa*) and the native accession ‘Kozjak’ (*Castanea sativa*) were analyzed. Endogenous indole-3-acetic acid (IAA) concentration was assessed at the beginning of propagation (day 0); in addition, strigolactones, flavonoids, rooting ability and quality were assessed 120 days after. The concentration of endogenous IAA in ‘Maraval’ (324.34 ± 28.66 ng g^−1^) and ‘Marsol’ (251.60 ± 35.44 ng g^−1^) was significantly higher than in ‘Kozjak’ (112.87 ± 35.44 ng g^−1^). The best rooting result was observed with the genotypes ‘Maraval’ (100.00 ± 0.00%) and ‘Marsol’ (90.48 ± 6.15%). A significantly lower strigol concentration was observed in the roots of ‘Maraval’ (75.54 ± 17.93 ng g^−1^) compared with other genotypes. The total flavonoid concentration in ‘Maraval’ was significantly higher (2794.99 ± 187.13 μg g^−1^) than in ‘Kozjak’ (1057.38 ± 61.05 μg g^−1^). Our results indicate that the concentration of endogenous IAA has a significant influence on rooting success. The results further indicate that in the case of flavonoids and strigolactones, not only the individual compounds but also their ratio is important for rooting success. Correlation coefficients calculated between analyzed compounds and rooting success point toward specific functions of flavonoids and strigolactones in the rooting of *Castanea* that need to be functionally analyzed.

## 1. Introduction

The genus *Castanea* belongs to the Fagaceae family and comprises seven species. The chestnut (*Castanea* sp.) is native to the temperate regions of the northern hemisphere and is cultivated for its nuts, beauty, shelter, and wood and has been an important part of the human diet [1]. The four most important species are the American chestnut (*Castanea dentata* Borkh. and *Castanea pumila* Mill.), Asian chestnuts (*Castanea mollissima* Blume, *Castanea henryi* Rehder & E.H. Wilson and *Castanea seguinii* Dode), Japanese chestnut (*Castanea crenata*) and European chestnut (*Castania sativa*). *Castanea sativa* is native to the Mediterranean countries of southern Europe and south-west Asia. The most important diseases of the tree are caused by some *Phytophthora* species and by chestnut blight (*Cryphonectria parasitica* M.E. Barr) [2,3]. Due to the massive decline in *Castanea sativa* in Europe, interspecific hybrids with Asian species have been increasingly bred. Interspecific hybrids are tolerant to American canker and chestnut blight, diseases that were previously responsible for the decline of the species [4,5]. Recent studies also report better rooting of interspecific hybrids [6,7,8,9].

The chestnut is a woody species known for its difficult generative or vegetative propagation [10]. Vegetative propagation by grafting is time-consuming or often fails due to incompatibility between scion and rootstock [1]. Propagation by cuttings is the most important and most frequently used method of vegetative propagation in horticulture. In this method, a crucial stage is the formation of adventitious roots [11]. The ability of plants to form adventitious roots on the basal part of stem cuttings varies greatly between different plant species and cultivars [12]. Adventitious root formation is often limited by the poor rooting ability of *Castanea* species [1].

Adventitious roots form from non-root tissue and can be the result of normal development or in response to stresses such as nutrient deficiency, flooding, heavy metal exposure, and biotic or abiotic wounding. The basis of cutting propagation is wound-induced adventitious rooting [13]. The formation of adventitious roots is a heritable quantitative genetic trait that is controlled by the interaction of several endogenous and environmental factors [14]. Exogenous stimuli that affect this process are light, wounding, temperature and mineral supply. The formation of adventitious roots is also regulated by endogenous factors, of which the phytohormone auxin is the most important plant hormone in root development [14].

Auxin metabolism genes regulate adventitious root formation by influencing auxin levels. Inhibition of polar auxin transport inhibits the early local increase in auxin, the induction of adventitious roots and the formation of sinks in the root regeneration zone [15]. Auxin can act synergistically or antagonistically with other hormones and other growth regulators such as cytokinins, brassinosteroids, ethylene, abscisic acid, gibberellins, jasmonic acid, polyamines and strigolactones to trigger cascades of events leading to adventitious root initiation and development [14,16]. The effect of IAA in the plant regarding the induction of root development is associated with a very short period immediately after the start of propagation (after cutting from a stock plant) [17]. Nag et al. [17] discovered that the presence of auxin in the base of the cutting is significant within 24 h after the start of propagation. An important mechanism for influencing auxin signaling pathways is amplification/inhibition by cofactors/inhibitors. Cofactors of auxin action often include phenolic substances [9]. It has been reported that substances, such as flavonoids, are associated with rooting [18]. It has been suggested that phenolics influence rooting by inhibiting IAA decarboxylation, which protects IAA from oxidation [19,20]. Increased flavonoid content could increase IAA concentration by regulating auxin transport, which is why easy-to-root genotypes were associated with higher flavonoid and endogenous IAA concentrations than difficult-to-root genotypes [12,21]. Previous reports indicate that the concentrations of endogenous phenolic compounds do not change in the days following severance [8,9,22].

Strigolactones, a group of phytohormones reported recently, have been characterized as inhibitors of adventitious root formation [16]. Strigolactones are produced primarily in roots [23]. Pacuar et al. [14] reported an inhibitory effect on adventitious root formation in *Arabidopsis* and *Pisum sativum*, as adventitious rooting was enhanced in strigolactones-deficient and response mutants. Negative regulation of strigolactones in adventitious root formation has been reported in both herbs and woody plants [24]. Inhibitory effects of strigolactones on the formation of adventitious roots were also reported by Fan et al. [24] in apple stem cuttings. Ozbilen et al. [25] reported on the effects of rac-GR24 as a strigolactone analog and TIS108 as a strigolactone inhibitor on easy and difficult-to-root olive cultivars, with the strigolactone inhibitor TIS108 increasing the rooting ability of cuttings of the difficult-to-root cultivar. The inhibitory effect of strigolactones on adventitious root formation appears to be indirect, mainly by inhibiting the positive role of auxin [26]. The quantification of specific strigolactones in plants could help to understand the role of this newly labeled group of plant hormones in adventitious root formation.

*Castanea sativa* is a widespread tree species in Slovenia. It covers more than 230,000 ha, which is 22% of the total forested area in Slovenia [3]. Hybrids are increasingly used because of their better rooting ability and their tolerance to common diseases, but also because of their interesting fruit for fruit growing. The conservation and propagation of Slovenian genotypes are essential for the preservation of biodiversity and the conservation of plant genetic resources in our area. Slovenian genotypes are adapted to our growing conditions; it is also important to preserve the natural and cultural heritage by preserving the genotypes native to the area. 

To obtain a first indication of whether endogenous concentrations of auxin, flavonoids and strigolactones may play a role in the low rooting of native genotypes of *Castanea*, we compared the rooting rate of native genotype ‘Kozjak’ and hybrid clones ‘Marsol’ and ‘Maraval’. Furthermore, the concentrations of endogenous auxin, flavonoids as cofactors and strigolactones as inhibitors for adventitious roots were analyzed and compared. This result should indicate whether these endogenous substances may limit the rooting of *Castanea*.

## 2. Results

### 2.1. Evaluation of the Root Development 

The best rooting result at the end of the rooting period was observed with the genotypes ‘Maraval’ (100.00 ± 0.00%) and ‘Marsol’ (90.48 ± 6.15%), while rooting success was significantly lower with the genotype ‘Kozjak’ (52.38 ± 14.29%), as shown in Table 1. Basal roots are roots that form at the base of the cutting. There was no difference in basal root formation between the genotypes. Acrobasal roots are roots that form at the base of the cutting and further up the cutting. No acrobasal root formation was observed in the ‘Kozjak’ genotype, while callus formation was observed in all evaluated cuttings of the ‘Kozjak’ genotype. Callus formation was significantly lower in ‘Maraval’ (58.33 ± 10.44%) and ‘Marsol’ (66.67 ± 00.00%). The number of main roots and the length of the root system were significantly higher in the ‘Maraval’ and ‘Marsol’ cuttings. The number of newly formed shoots was significantly higher in the ‘Marsol’ genotype than in ‘Kozjak’, while the length of newly formed shoots differed between all genotypes (see Table 1).

### 2.2. Indole-3-Acetic Acid (IAA) Quantification

The endogenous IAA concentration was also analyzed in the stem base (3 cm) of the freshly harvested cuttings (day 0) of all genotypes evaluated (see Table 2). A significantly lower endogenous IAA concentration was found in the genotype ‘Kozjak’ (112.87 ± 35.44 ng g^−1^) than in genotypes ‘Marsol’ (251.60 ± 35.44 ng g^−1^) and ‘Maraval’ (324.34 ± 28.66 ng g^−1^).

### 2.3. Strigolactones Quantification

The concentration of strigolactones was evaluated in the roots and callus of the cuttings of the genotypes ‘Maraval’, ‘Marsol’ and ‘Kozjak’ at the end of the rooting period (see Table 3). No differences in orobanchol concentration were found between the genotypes and the material (callus, roots) used. A significantly lower strigol concentration was found in the roots of ‘Maraval’ (75.54 ± 17.93 ng g^−1^) compared with the roots of ‘Kozjak’ (164.05 ± 20.71 ng g^−1^) and ‘Marsol’ (152.03 ± 20.71 ng g^−1^). Strigol concentration in ‘Kozjak’ callus was significantly lower (75.31 ± 17.93 ng g^−1^) than in ‘Marsol’ callus (145.32 ± 17.93 ng g^−1^). There was no difference in strigol concentration between the roots and callus of ‘Marsol’ and ‘Maraval’, while the strigol concentration in the ‘Kozjak’ genotype was significantly higher in the roots (164.05 ± 20.71 ng g^−1)^ than in the callus (75.31 ± 17.93 ng g^−1^). The only difference in 5-deoxystrigol concentration was observed between the roots of ‘Kozjak’(28.97 ± 13.30 ng g^−1^) and ‘Marsol’ (107.63 ± 28.65 ng g^−1^). There were no differences in the total concentration of strigolactones between the genotypes in callus or roots, while the ratio of individual strigolactones in the genotypes analyzed was different (see Supplementary Material, Figure S1). 

Strigol concentration in the roots was negatively and moderately correlated with the length of newly formed shoots, the number of main roots, and the length of the root system, and it was positively and moderately correlated with callus formation (Table 4). Orobanchol concentration in the roots of *Castanea* sp. is negatively and moderately correlated with the length of the newly formed shoots, the number of main roots and the length of the root system, as well as the successful rooting of the cuttings. No moderate or higher correlation was observed between 5-deoxy-strigol and the quality of the cuttings (number of newly formed shoots, length of newly formed shoots, number of main roots, length of the root system, callus formation, acrobasal rooting, basal rooting, successfully rooted cuttings) (see Supplementary Material, Table S4). For the selected genotypes, a positive moderate correlation was found between the strigol concentration in the callus and the number of newly formed shoots, the length of newly formed shoots and the length of the root system (see Supplementary Material, Table S5).

### 2.4. Flavanoids Quantification

**Figure 1:**
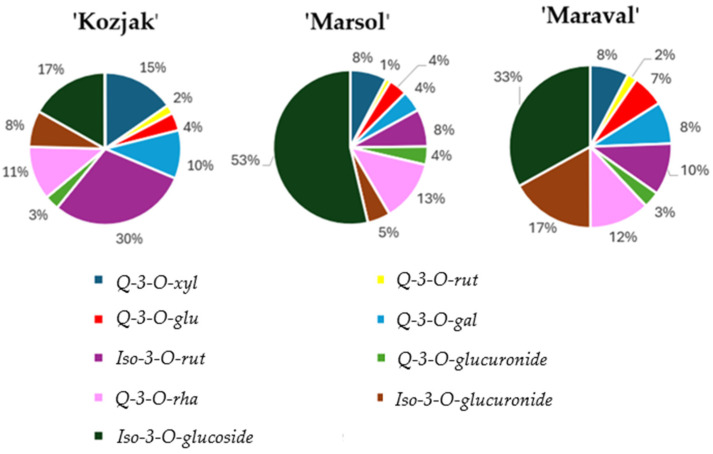
Percentage of flavonoid compounds in shoots of ‘Kozjak’, ‘Marsol’ and ‘Maraval’. Q-3-O-xyl: Quercetin-3-O-xyloside; Q-3-O-rut: Quercetin-3-O-rutinoside; Q-3-O-glu: Quercetin-3-glucoside; Q-3-O-gal: Quercetin-3-O-galactoside; Iso-3-O-rut: Isorhamnetin-3-O-rutinoside; Q-3-O-glucuronide: Quercetin-3-O-glucuronide; Q-3-O-rha: Quercetin-3-O-rhamnoside; Iso-3-O-glucuronide: Isorhamnetin-3-O-glucuronide; Iso-3-O-glucoside: Isorhamnetin 3-O-glucoside.

Differences in flavonoid compounds between ‘Kozjak’ and ‘Maraval’ were found for all identified compounds except for isorhamnetin-3-O-rutinoside (see Table 4), the latter accounting for 30.00% of all flavonoid compounds in ‘Kozjak’ and 10.00% in ‘Maraval’ (see Figure 1). In ‘Marsol’, the concentrations of quercetin-3-O-rutinoside, quercetin-3-O-glucoside, quercetin-3-O-galactoside, isorhamnetin-3-O-rutinoside and isorhamnetin-3-O-glucuronide were significantly lower than in the ’Maraval’ genotype. The isorhamnetin-3-O-glucoside concentration in ‘Marsol’ was significantly higher than in ‘Maraval’. The concentrations of quercetin-3-O-glucoside, isorhamnetin-3-O-rutinoside, quercetin-3-O-glucuronide, quercetin-3-O-rhamnoside and isorhamnetin-3-O-glucoside were different in the ‘Kozjak’ and ‘Marsol’ genotypes. The differences between the genotypes were found not only in the concentration of the identified flavonoids but also in their ratio (see Figure 1). 

### 2.5. Correlation between Auxin, Flavonoid Concentration and Root Development

Flavonoid concentration in stem cuttings and root development were evaluated and analyzed at the end of the rooting period (day 120). The number of newly formed shoots was positively moderately correlated with Iso-3-O-glucoside concentration, the length of newly formed shoots was positively moderately correlated with Q-3-O-xyl and Q-3-O-glucuronide concentration and positively highly correlated with Q-3-O-rut, Q-3-O-glu, Q-3-O-gal, Q-3-O-glucuronide, Q-3-O-rha and Iso-3-O-glucoside concentration (see Table 5). The number of main roots correlated positively moderately with Iso-3-O-glucuronide and Q-3-O-galactoside concentrations and positively highly correlated with Q-3-O-rut, Q-3-O-glu, Q-3-O-glucuronide, Q-3-O-rha and Iso-3-O-glucoside concentrations. The length of the root system was positively moderately correlated with the concentration of Q-3-O-gal and negatively moderately correlated with the concentration of Iso-3-O-rutinoside. Callus formation was negatively moderately correlated with the concentration of Q-3-O-rut, Q-3-O-glu, Q-3-O-gal, Q-3-O-glucuronide, Q-3-O-rha and Iso-3-O-glucuronide. Acrobasal rooting correlated positively and moderately with the concentration of Q-3-O-rut, Q-3-O-glucoside, Q-3-O-glucuronide, Q-3-O-rha, Iso-3-O-glucoside and Iso-3-O-glucuronide. Successful rooting correlated positively and moderately with the concentration of Q-3-O-rut, Iso-3-O-glucuronide, Q-3-O-glu, and Q-3-O-glucuronide and highly positively correlated with the concentration of Q-3-O-rha and Iso-3-O-glucoside. 

The IAA concentration was analyzed in the stem base at day 0 and was positively highly correlated with the length of newly formed shoots and positively, moderately correlated with the number of newly formed shoots, the number of main roots, acrobasal rooting and successfully rooted cuttings (see Supplementary Material, Table S3).

## 3. Discussion

Many authors reported difficulties in the root formation of *Castanea sativa* [10,27,28] under in vivo and in vitro conditions. Our results also showed the difficulty in the rooting of *Castanea sativa* (‘Kozjak’) with significantly lower rooting success and higher undesirable traits compared with the Japanese × European chestnut hybrids (*Castanea crenata* × *Castanea sativa)* ’Marsol’ and ‘Maraval’. Callus development is usually the result of difficult rooting [11], which we were also able to confirm with our results. Callus development was observed in all evaluated cuttings of the ‘Kozjak’ genotype, while callus development was significantly lower in ‘Marsol’ and ‘Maraval’. According to Kunc et al. [11], acrobasal roots are more desirable in horticultural practice because the rooted cuttings are easier to dig up, and the newly formed roots are less damaged during digging and transplanting. The main root number is also a very good indicator of root quality [7]. Our results are in agreement with those of Kunc et al. [11], who report a lower proportion of acrobasal roots in cuttings obtained from mature stock material (difficult-to-root) of *Prunus subhirtella* Miq. ‘Autumnalis’ and lower rooting results compared with cuttings obtained from rejuvenated stock material (easy-to-root). Eugenia Miranda-Fontaina [29] also reported more undesirable characteristics of *Castanea sativa* compared with hybrid clones. In contrast to our results, Osterc et al. [8] reported differences in rooting and other evaluation parameters between the ‘Maraval’ and ‘Marsol’ genotypes. According to their results, the ‘Maraval’ genotype rooted better, the root system was longer, and the number of main roots was greater. Osterc et al. [9] and Stefanic et al. [30] also reported better rooting and less frequent callus formation in the ‘Maraval’ compared with the ‘Marsol’ genotype. In the results of Song et al. [6], no differences in rooting success were found between ‘Marsol’ and ‘Maraval’, which is consistent with our results. High endogenous auxin concentration in the induction phase (0–24 h) of adventitious root formation is associated with a high rooting rate in *Sequoia sempervirens* Endl. [31] and *Prunus dulcis* Batsch. [32]. These results are consistent with our results, where the genotype ‘Maraval’ with the highest endogenous IAA concentration in the induction phase also had the highest rooting rate (see Table 1 and Table 2 and Supplementary Material Table S3). Kunc et al. [11] reported that the uptake of exogenously applied indole-3-butyric-acid (IBA) does not differ between easy and difficult-to-root plant material. With the same addition of IBA, the total auxin content increases equally for easy and difficult-to-root plant material. The present results highlight the importance of endogenous auxin for adventitious root induction in *Castanea*. 

Even though the strigolactones and flavonoids were measured at the end of the rooting period, their concentrations may indicate the functions of these compounds in the rooting process. Strigolactones are a special class of phytohormones that are categorized as repressors of adventitious root formation. However, the role of specific strigolactones is poorly understood [33]. The concentration of three strigolactones (strigol, orobanchol and 5-deoxystrigol) in the roots and callus of three genotypes of *Castanea* sp. (‘Kozjak’, ‘Marsol’, ‘Maraval’) and their correlation with initial endogenous IAA in the stem base were evaluated (see Supplementary Material, Table S2). Previous studies [33,34,35,36] identified several genes of the strigolactone biosynthic pathway and showed that the inhibition of strigolactones promotes adventitious root formation. Our results may indicate that strigol and orobanchol in roots were negatively correlated with rooting parameters, while 5-deoxystrigol has no inhibitory effect on rooting parameters (see Supplementary Material, Table S4). As can be seen from Figure S1, 5-deoxystrigol is represented to a greater extent in the genotypes with higher rooting rates, ‘Marsol’ and ‘Maraval’. Our results may indicate that the group of strigolactones does not only have an inhibitory effect on adventitious root development. There are no significant differences in the total strigolactone concentration, although the rooting result is different between genotypes. This may be attributed to the fact that individual strigolactones have different effects on the rooting process. Sun et al. [37] also reported that strigolactones can positively regulate the growth of adventitious roots. There is no evidence for the role of specific strigolactone in adventitious root formation. Given the lack of research on strigolactones, the identification of the specific strigolactones synthesized in plant roots is crucial. According to previous studies [16,17,18,19], we expected a higher concentration of strigolactones in the ‘Kozjak’ genotype, which showed the lowest rooting results and, consequently, many undesirable traits. The strigol concentration in the roots of ‘Kozjak’ and ‘Marsol’ was significantly higher than in ‘Maraval’. This result agrees with previous studies [12,13,14] indicating better rooting of ‘Maraval’ compared with ‘Marsol’. This difference could also be explained by the ratio of strigolactones. The ratio of strigolactones in ‘Marsol’ and ‘Maraval’ is similar, with the strigol content being low and the 5-deoxystrigol content being high, which could indicate a positive effect of the latter on the formation of adventitious roots. Orobanchol in roots is negatively correlated with the development of adventitious roots, but there are no differences in concentration between genotypes. It can, therefore, be concluded that orobanchol does not play an important role as an inhibitor of adventitious root formation. A positive moderate correlation between IAA and orobanchol was observed (see Supplementary Material, Table S2). Previous studies [16,37,38,39] reported a negative interaction between strigolactones and auxins in relation to root system formation, branching and other functions of strigolactones in the plant. Furthermore, the effects of strigolactone on root growth are likely linked to root responses not only to auxin but also to other plant hormones that influence root architecture. Understanding the role of individual strigolactones in the formation of adventitious roots is the key to the first step in achieving better rooting in difficult-to-root species.

The results of the flavonoid analyses showed differences in the concentration of flavonoid compounds not only between ‘Kozjak’ and ‘Maraval’ but also between ‘Marsol’ and ‘Maraval’. The correlation between the IAA concentration and the flavonoids was also analyzed (see Supplementary Material, Table S1). Since there were no significant differences in the evaluation of rooting between ‘Marsol’ and ‘Maraval’, it can be concluded that it is not only the concentration of individual flavonoids that is decisive for adventitious root formation but also the total concentration and ratio. These results agree with recent studies [12,19,40], which report that a higher concentration of total flavonoids also had a positive effect on rooting results. Higher concentrations of total flavonoids were observed in easy-to-root genotypes than in difficult-to-root genotypes. Osterc et al. [8] also reported a highly significant correlation between quercetin concentrations and the rooting process. Zanoni do Prado et al. [18] reported that substances such as flavonoids are associated with rooting. The concentration of quercetin-3-O-glucoside and quercetin-3-O-rhamnoside was determined in cuttings of *Eucalyptus gunnii* Hook.f.. The two identified flavonoids were present in very low amounts in cuttings that could not root and in higher amounts in cuttings that could root, which is consistent with our results. 

The significance of the proportion of the individual flavonoids in adventitious root formation has not been investigated yet. Since the ratio of specific flavonoids varied among the genotypes analyzed in our study, a correlation test was performed to investigate the relationship between the specific flavonoids and the rooting process. In ‘Kozjak’, ‘Marsol’ and ‘Maraval’, the main differences were observed in the distribution of isorhamnetin-3-O-glucoside, isorhamnetin-3-O-glucuronide and isorhamnetin-3-O-rutinoside. The percentage of isorhamnetin-3-O-glucoside in ‘Marsol’ was 53.00%, 33.00% in ‘Maraval’ and 17.00% in ‘Kozjak’. The percentage of isorhamnetin-3-O-glucuronide in ‘Marsol’ was 5.00%, 17.00% in ‘Maraval’ and 8.00% in ‘Kozjak’. The percentage of isorhamnetin-3-O-rutinoside was 8.00% in ‘Marsol’, 10.00% in ‘Maraval’ and 30.00% in ‘Kozjak’ (Figure 1).

No significant differences in isorhamnetin-3-O rutinoside concentration were found between ‘Kozjak’ and ‘Maraval’, but the proportion in ‘Kozjak’ was higher (30.00%) than in ‘Marsol’ and ‘Maraval’ (8.00 and 10.00%). Since isorhamnetin-3-O-rutinoside was moderately and negatively correlated with the length of the root system, a significantly shorter length of the root system in the ‘Kozjak’ genotype could be explained by a higher proportion of isorhamnetin-3-O-rutinoside. The proportion and concentration of isorhamnetin-3-O-glucuronide were similar in ‘Kozjak’ and ‘Marsol’ but significantly higher in ‘Maraval’. Isorhamnetin-3-O-glucuronide correlated moderately with the number of main roots. The number of main roots also correlated with isorhamnetin-3-O-glucoside, but the correlation coefficient was high. The higher number of main roots in ‘Marsol’ can be explained by the high correlation between isorhamnetin-3-O-glucoside and the number of main roots, including a higher proportion of isorhamnetin-3-O-glucoside in ‘Marsol’ than in ‘Kozjak’.

Isorhamnetin-3-O-glucuronide and isorhamnetin-3-O-glucoside correlated highly with the length of newly formed shoots. The ratio of isorhamnetin-3-O-glucuronide in ‘Marsol was 5.00% and 8.00% in ‘Kozjak’, while the isorhamnetin-3-O-glucoside ratio was 53.00% in ‘Marsol’ and 17.00% in ‘Kozjak’, thus explaining the longer newly formed shoots in ‘Marsol’. The concentration of isorhamnetin-3-O-glucoside, which is associated with successful rooting, the number of main roots and the length of newly formed shoots, length of newly formed shoots and acrobasal rooting, is significantly different between all genotypes. A higher proportion of isorhamnetin-3-O-glucoside in ‘Maraval’ and ‘Marson’ is manifested in lower rooting success, the number of main roots and acrobasal root formation in ‘Kozjak’.

## 4. Material and Methods

### 4.1. Samples

Leafy cuttings of the two commercial cultivars ‘Marsol’ and ‘Maraval’ (*Castanea crenata* × *Castanea sativa*) and native accession ‘Kozjak’ (*Castanea sativa*) were collected in June from an Experimental Field for Nut Crops in Maribor (Slovenia; 46°34′01″ N; 15°37′51″ E; 280 m a.s.l.). 

### 4.2. Experimental Design 

The shoots of the current year of each genotype (*n* = 15) were used for the auxin analyses on the day the experiment was set up. The shoots were removed from the stock plant, and from the lowest parts, 3 cm of the cuttings were placed in liquid nitrogen, freeze-dried using the lyophilization method and stored for the analysis of endogenous IAA. 

The propagation beds in which the cuttings were placed were divided into 15 plots (40 × 40 cm), with five replicates for each genotype and three biological replicates in each plot (*n* = 15). After preparation, the leafy cuttings were planted in the plots for 120 days. The quality of the cuttings was evaluated in autumn, at the end of the growing season in October. The samples were washed so that no residues of substrate or IBA mixture were present. Root formation and quality of cuttings were assessed according to the protocol described by Kunc et al. [11]. Callus, roots, and shoots were separated, placed in liquid nitrogen, freeze-dried using the lyophilization method, and stored at −20 °C for further analyses. In the next steps, the concentration of strigolactones (strigol, orobanchol, 5-deoxystrigol) in the callus and roots was analyzed. The identification and quantification of flavonoid compounds in the shoots of selected genotypes were also carried out. The experiment was set up as a single factorial design with five replicates. The distribution of replicates was based on simple random sampling. Each plot represented a single experimental replicate. 

### 4.3. Preparation of the Cuttings

The shoots of the current year of each genotype were used as plant material for the experiment. The cuttings were shortened to a length of 15 cm, and the lower four leaves were removed. The base of the cuttings was treated with 0.0025 M IBA using the quick dip method (the basal end of 0.5 to 1 cm of the cutting is dipped in the solution for 3 to 5 s) and placed in the substrate. The substrate used was a mixture of peat and sand in a 1:1 ratio and was fertilized with 2.0 g/L 3–4 M Osmocote Exact fertilizer (16 (N)-9 (P)-12 (K) + 2(MgO) + trace elements) (ICL). The experiment was carried out in a greenhouse at the Biotechnical Faculty. The greenhouse was equipped with an automatically controlled fogging system that enabled a humidity of 98–100%. A high-pressure fogging system (Plantog, Fischament, Austria), which enables a high-quality rooting process, was used. The air temperatures in the greenhouse fluctuated greatly throughout the day, up to 50 °C during the day and between 18 and 20 °C at night. The system was in operation from 8 a.m. to 8 p.m. and was switched off at certain intervals. On sunny days, the interval was set to 40 s of fogging and a 1 min pause. On cloudy and rainy days, the intervals were extended to 3 min. The fogging system was always switched off at night. The experiment was conducted for 120 days, from June 2023 to October 2023.

### 4.4. Indole-3-Acetic Acid (IAA) Analyses

IAA was extracted according to the modified method of Trapp et al. [41]. A total of 100 mg of dry samples (the lowest parts, 3 cm of the cuttings) were ground to a fine powder using a mortar and pestle in liquid nitrogen. Extraction was performed, adding 1.0 mL of isopropanol into each tube containing dry plant material. Samples were shaken (Unimax 1010 shaker, Heidolph, Schwabach, Germany) for 30 min centrifuged at 11,000× *g* and 4° C for 5 min. The supernatant was transferred into a new 2 mL Eppendorf tube and dried in speed vac (Eppendorf). After drying, 200 µL of MeOH was added to each sample, homogenized and centrifuged at 11,000× *g* and 4 °C for 10 min. 

The supernatant was analyzed using a UHPLC system (Dionex UltiMate 3000; Thermo Scientific, Waltham, MA, USA) coupled with a tandem mass spectrometer (LTQ XL; Thermo Scientific, Waltham, MA, USA). The C18 column (150 × 4.60 mm; 3 µm column; Gemini; Phenomenex, Torrance, CA, USA) was used. The temperature was operated at 25 °C. The solvents used for the analysis were solvent A, consisting of 0.1% formic acid with 3% acetonitrile in double-distilled water (*v*/*v*/*v*), and solvent B, consisting of 0.1% formic acid with 3% double-distilled water in acetonitrile (*v*/*v*/*v*). The elution profile was as follows: 0–10 min, 42–55% B; 10–13 min, 55–100% B; 13–15 min 100% B; 15.1–20 min 42% B; and at 20–23 min, the reconditioning of the column was performed. The mobile phase flow rate was 0.6 mL min^−1^. The injection volume was 20 µL. The identification of IAA was achieved by coupling the UHPLC system with a tandem mass spectrometer (LTQ XL; Thermo Scientific, Waltham, MA, USA) using heated electrospray ionization. The mass spectrometer was operated in negative ion mode. The following parameters were used: capillary temperature 200 °C; capillary voltage 8.0 V; sheath temperature 200 °C; sheath gas 30 arb; ion spray voltage 4.5 kV; tube lens −82 V; and auxiliary gas 10 arb. After determining the retention time and identifying typical fragmentation patterns of the obtained quantification standard, Selected Reaction Monitoring (SRM) was used to identify the selected compound. The transitions with the optimized parameters and retention times are shown in Table 6.

### 4.5. Strigolactones Analyses

Strigolactones were extracted according to the modified method by López-Ráez et al. [42] and Floková et al. [43], in which 100 mg of dry tissue was ground in a mortar with liquid nitrogen. The samples were extracted for 60 min in a 10 mL glass vial with 1.5 mL of cold ethyl acetate. The samples were then shaken in ice-cold water (Unimax 1010 shaker, Heidolph, Schwabach, Germany). The samples were centrifuged at 2000× *g* at 4 °C for 15 min. The organic phase was then carefully transferred to a 2 mL Eppendorf tube. The pellets were re-extracted with another 0.5 mL ethyl acetate; then, the combined ethyl acetate fractions were dried using the speed vac (Eppendorf). The dry residue was dissolved in 1 mL of 10% aqueous acetonitrile. The extracts were further pre-concentrated using reversed-phase polymeric Oasis^®^ HLB columns (150 mg/3 mL, Waters, Milford, MA, USA). The sorbent in the columns was activated with 3 mL of 100% acetonitrile and equilibrated with 10% aqueous acetonitrile. The samples of the tissue extracts were passed through the activated column. A further 3 mL of 10% aqueous acetonitrile was added. The strigolactone analytes were eluted from the column with 1 mL of 80 aqueous acetone. The solution was filtered into the vials, and the samples were stored at −20 °C until further analysis.

Phytohormones were analyzed on a UHPLC system (Dionex UltiMate 3000; Thermo Scientific, Waltham, MA, USA) coupled to a tandem mass spectrometer (LTQ XL; Thermo Scientific, Waltham, MA, USA) following the modified method by Almeida Trapp et al. [41]. The C18 column (150 × 4.60 mm; 3 µm column; Gemini; Phenomenex, Torrance, CA, USA) was used for the separation of strigol, orobanchol and 5-deoxystrigol. The temperature was set to 50 °C. The solvents used for the analysis were solvent A, comprising 0.1% formic acid with 3% acetonitrile in double distilled water (*v*/*v*/*v*), and solvent B, comprising 0.1% formic acid with 3% double distilled water in acetonitrile (*v*/*v*/*v*). The elution profile was 0–1 min, 0–25% B; 1.1–7.5 min, 25–45% B; 7.6–12 min, 45–70% B; 12–15 min, 70–10% B; and 15–18 min, 10–0% B. The mobile phase flow rate was 0.4 mL min^−1^, and the injection volume was 20 µL. The phytohormones were identified by coupling the UHPLC system with a tandem mass spectrometer (LTQ XL; Thermo Scientific, Waltham, MA, USA) using heated electrospray ionization. The mass spectrometer was operated in a positive ion mode to identify the strigolactones. To identify the strigolactones, the following parameters were used: capillary temperature 200 °C, capillary voltage 8.0 V, sheath temperature 280 °C, sheath gas 35 arb, ion spray voltage 4.5 kV, tube lens 45 V and auxiliary gas 5 arb. Selected Reaction Monitoring (SRM) was used to identify these selected compounds after determining the retention time and identifying typical fragmentation patterns of the obtained quantification standard. The transitions with the optimized parameters and retention times are listed in Table 6.

### 4.6. Flavonoid Analyses

The phenolic compounds were extracted according to the protocol described by Medic et al. [44]. A total of 100 mg of the freeze-dried sample was extracted with 2 mL of 80% methanol in double-distilled water. The extraction ratio was 1:20 (*w*/*v*). The samples were then shaken (TOP-MIX 94500 vortex mixer; Heidolph, Schwabach, Germany), sonicated for 60 min in ice water (Sonis 4 ultrasonic bath; Iskrapio, Slovenia) and centrifuged at 10,000× *g* at 4 °C for 10 min. The samples were filtered with a 0.2 μm polyamide filter (Chromafil AO 20/25; Macherey-Nagel, Düren, Germany) and stored at −20 °C for further analysis. An LTQ XL tandem mass spectrometer with heated electrospray ionization in negative mode connected to a Vanquish UHPLC system (Thermo Scientific, Waltham, MA, USA) with a diode array detector at 350 nm was used to identify and quantify the flavonoids. All parameters were used as described by Medic et al. [44]. The flavonoid compounds identified in *Castanea* sp. are listed in Table 7 and Figure S2. The quantification of the different flavonoid compounds is given in Table 2. The individual compounds were separated by HPLC and identified by a comparison with internal standards. All parameters were used as described by Medic et al. [44].

### 4.7. Chemicals 

The standards used to determine the phenolic compounds in the samples were quercetin-3-O-galactoside, quercetin-3-O-glucoside, quercetin-3-O-rhamnoside and quercetin-3-O-rutinoside from Sigma-Aldrich Co. (St. Louis, MO, USA). Isorhamnetin-3-O-glucoside was purchased from Extrasynthese (Genay, France) and quercetin-3-O-glucuronide, quercetin-3-O-xyloside, isorhamnetin-3-O-rutinoside and isorhamnetin-3-O-glucuronide were purchased from Fluka Chemie (Steinhein, Germany).

The following standards were used to identify and quantify the phytohormones: orobanchol, 5-deoxystrigol and strigol (Sigma-Aldrich Chemie GmbH, Steinheim, Germany). For extraction and further analysis, we used formic acid, ethyl acetate and acetonitrile (Sigma-Aldrich Chemie GmbH, Steinheim, Germany).

Acetonitrile for the mobile phases was HPLC-MS grade (Fluka Chemie GmbH, Buchs, Switzerland). Methanol and formic acid were HPLC grade (Sigma-Aldrich Chemie GmbH, Steinheim, Germany). The bi-distilled water was purified using a water purification system (Milli-Q, Millipore, Bedford, MA, USA).

### 4.8. Statistical Analysis

The data were collected with Microsoft Excel 2016 and statistically analyzed with R commander (package Rcmdr) version 4.1.0. A one-way analysis of variance (ANOVA) was performed. The “emmeans” package and Duncan tests were used for pairwise post hoc comparisons. A residual plot was used to analyze the normality and homoscedasticity of the data. All data were presented as means ± standard error (SE). Statistical means were calculated at a confidence level of 95% to determine the significance of differences (*p* < 0.05).

## 5. Conclusions

In our research, we focused on the biochemical differences between the *Castanea sativa* genotype and the chestnut hybrid genotypes (*Castanea crenata* × *Castanea sativa*). Our research confirmed that rooting success is related to the endogenous concentration of endogenous IAA. Genotypes with a lower endogenous IAA concentration at the time of cutting excision had a lower rooting rate. *Castanea crenata* × *Castanea sativa* hybrids have significantly higher rooting rates and lower callus formation than ‘Kozjak’ (*Castanea sativa*). Our results suggest that the individual and total flavonoids contribute not only to the formation of the adventitious system but also to the ratio between them, which differs between genotypes. When analyzing the strigolactones, the results showed that strigol and orobanchol were negatively correlated to the rooting parameters, while 5-deoxystrigol was not related to the formation of adventitious roots. The data indicated that not only the individual strigolactone compounds but also their ratio is important for rooting success. The role of the individual strigolactones is crucial for a better understanding of the role of strigolactones in adventitious rooting. The exogenous addition of individual strigolactones would confirm their role in adventitious root formation. 

## Figures and Tables

**Table 1:** Evaluation of the newly formed adventitious root system (day 120) according to genotype.

**Genotype**	**Successfully Rooted Leafy Cuttings ± SE (%)**	**Cuttings with Basal Root Development ± SE (%)**	**Cuttings with Acrobasal Root Development ± SE (%)**	**Cuttings with Callus Formation ± SE (%)**
‘Marsol’	90.48 ± 6.15 **b**	52.38 ± 14.28 **a**	33.33 ± 8.90 **b**	66.67 ± 00.00 **a**
‘Maraval’	100.00 ± 0.00 **b**	66.67 ± 8.91 **a**	38.10 ± 11.34 **b**	58.33 ± 10.44 **a**
‘Kozjak’	52.38 ± 14.29 **a**	52.38 ± 6.74 **a**	00.00 ± 00.00 **a**	100.00 ± 00.00 **b**
**Genotype**	**Number of main roots ± SE**	**Length of the root system ± SE (cm)**	**Length of the newly formed shoots ± SE (cm)**	**Number of the newly formed shoots ± SE**
‘Marsol’	5.14 ± 0.48 **b**	9.24 ± 0.94 **b**	12.60 ± 0.82 **b**	2.14 ± 0.30 **b**
‘Maraval’	4.42 ± 0.26 **b**	10.75 ± 0.92 **b**	19.12 ± 2.73 **c**	1.50 ± 0.06 **ab**
‘Kozjak’	1.23 ± 0.33 **a**	4.50 ± 1.04 **a**	1.53 ± 0.37 **a**	1.10 ± 0.22 **a**

The different bolded lowercase letters in the same columns indicate a significant difference (*p* < 0.05) (ANOVA, *n* = 15).

**Table 2:** Concentration of endogenous IAA (day 0) according to genotype.

Genotype	IAA Concentration (ng g^−1^ DW ± SE)
‘Marsol’	251.60 ± 35.44 **b**
‘Maraval’	324.34 ± 28.66 **b**
‘Kozjak’	112.87 ± 35.44 **a**

DW: dry weight. The different bolded lowercase letters in the same columns indicate a significant difference (*p* < 0.05) (ANOVA, *n* = 15).

**Table 3:** Strigolactone concentration (day 120) according to genotype and type of plant material.

Genotype	Type of Material	Orobanchol ± SE (ng g^−1^ DW)	Strigol ± SE (ng g^−1^ DW)	5-Deoxystrigol ± SE (ng g^−1^ DW)	TSC (ng g^−1^ DW)
‘Marsol’	Roots	44.03 ± 3.49 **a**	152.03 ± 20.71 **b**	107.63 ± 28.65 **b**	303.69 ± 52.85 **b**
‘Marsol’	Callus	44.77 ± 11.16 **a**	145.32 ± 17.93 **b**	57.74 ± 14.65 **ab**	250.83 ±43.74 **ab**
‘Maraval’	Roots	22.12 ± 1.55 **a**	75.54 ± 17.93 **a**	91.40 ± 33.92 **ab**	188.06 ± 53.40 **ab**
‘Maraval’	Callus	28.36 ± 7.79 **a**	130.04 ± 33.10 **ab**	76.22 ± 20.38 **ab**	234.62 ± 61.27 **ab**
‘Kozjak’	Roots	41.93 ± 6.35 **a**	164.05 ± 20.71 **b**	28.97 ± 13.30 **a**	234.95 ± 40.36 **ab**
‘Kozjak’	Callus	24.01 ± 3.13 **a**	75.31 ± 17.93 **a**	47.13 ± 19.27 **ab**	146.45 ± 40.33 **a**

TSC: Total strigolactone concentration. DW: dry weight. The different bolded lowercase letters in the same columns indicate a significant difference (*p* < 0.05) (ANOVA, *n* = 15).

**Table 4:** Flavonoid compounds in stem cuttings (day 120) of genotypes ‘Kozjak’, ‘Maraval’ and ‘Marsol’.

Flavonoid	‘Kozjak’ ± SE (μg g^−1^ DW)	‘Maraval’ ± SE (μg g^−1^ DW)	‘Marsol’ ± SE (μg g^−1^ DW)
Quercetin-3-O-rutinoside	19.95 ± 1.21 **a**	53.04 ± 3.01 **b**	22.89 ± 0.86 **a**
Quercetin-3-O-glucoside	40.36 ± 1.45 **a**	179.77 ± 10.57 **c**	88.66. ± 3.99 **b**
Quercetin-3-O-galactoside	108.88 ± 6.45 **a**	233.52 ± 5.95 **b**	101.30 ± 4.42 **a**
Isorhamnetin-3-O-rutinoside	312.51 ± 18.34 **b**	288.84 ± 14.72 **b**	180.11 ± 16.94 **a**
Quercetin-3-O-glucuronide	32.65 ± 1.34 **a**	90.77 ± 5.29 **b**	87.75 ± 12.02 **b**
Quercetin-3-O-rhamnoside	121.12 ± 4.55 **a**	334.70 ± 18.17 **b**	296.82 ± 19.58 **b**
Isorhamnetin-3-O-glucuronide	81.76 ± 4.43 **a**	475.11 ± 28.27 **b**	110.07 ± 9.38 **a**
Isorhamnetin-3-O-glucoside	177.40 ± 7.17 **a**	923.50 ± 90.08 **b**	1233.17 ± 68.52 **c**
Quercetin-3-O-xyloside	162.75 ± 16.11 **a**	215.74 ± 11.07 **b**	177.30 ± 9.62 **ab**
Total flavonoid concentration	1057.38 ± 61.05 **a**	2794.99 ± 187.13 **c**	2298.07 ± 143.33 **b**

DW: dry weight. Data are means ± standard error. The different bolded lowercase letters in the same rows indicate a significant difference (*p* < 0.05) (ANOVA, *n* = 15).

**Table 5:** Correlation coefficients between flavonoid concentration in stem cuttings and root development (day 120).

	Q-3-O-xyl	Q-3-O-rut	Q-3-glucoside	Q-3-O-gal	Iso-3-O-rut	Q-3-O-glucuronide	Q-3-O-rha	Iso- 3-O-glucuronide	Iso-3-O-glucoside
Number of newly formed shoots	0.08	−0.01	0.14	−0.09	−0.39	0.29	0.34	−0.03	0.54
Length of newly formed shoots	0.48	0.74	0.88	0.60	−0.01	0.56	0.81	0.77	0.64
Number of main roots	0.34	0.62	0.69	0.48	−0.17	0.64	0.74	0.58	0.62
Root system length	0.00	0.26	0.17	0.42	−0.40	0.37	−0.06	0.16	−0.06
Callus formation	−0.07	−0.44	−0.45	−0.49	0.36	−0.46	−0.40	−0.44	−0.30
Acrobasal rooting	0.22	0.44	0.48	0.31	−0.22	0.51	0.58	0.40	0.50
Basal rooting	0.08	−0.02	0.10	−0.03	0.07	−0.07	0.06	0.03	0.11
Successfully rooted	0.30	0.41	0.57	0.28	−0.15	0.43	0.63	0.43	0.61
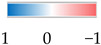									

Q-3-O-xyl: Quercetin-3-O-xyloside; Q-3-O-rut: Quercetin-3-O-rutinoside; Q-3-O-glu: Quercetin-3-glucoside; Q-3-O-gal: Quercetin-3-O-galactoside; Iso-3-O-rut: Isorhamnetin-3-O-rutinoside; Q-3-O-glucuronide: Quercetin-3-O-glucuronide; Q-3-O-rha: Quercetin-3-O-rhamnoside; Iso-3-O-glucuronide: Isorhamnetin-3-O-glucuronide; Iso-3-O-glucoside: Isorhamnetin 3-O-glucoside. The scale of Pearson’s Correlation Coefficient: 0–0.19: very low correlation; 0.2–0.39: low correlation; 0.4–0.59: moderate correlation; 0.6–0.79: high correlation; 0.8–1: very high correlation. (Correlation matrix, *n* = 15).

**Table 6:** SRM transitions for phytohormone quantifications.

Phytohormone	Retention Time	Pseudo-Molecular Ions *(m*/*z)*		Fragmentation Pattern
	(min)	${\left[M+H\right]}^{+}$	${\left[M-H\right]}^{-}$	(Relative Peak Intensity %)
IAA	5.50	/	174	130 (100)
Orobanchol	15.76	347		329 (100), 233 (86), 205 (8), 303 (6), 257(6)
Strigon	15.70	345		231 (100), 327 (27), 249 (21)
5-deoxystrigol	14.50	331		313 (100), 234 (47)
Strigol	15.75	347		233 (100), 329 (47), 163 (17), 251 (15), 215 (11)

IAA: Indole-3-acetic acid, [M − H]^−^, pseudo-molecular ion identified in negative ion mode, [M + H]^+^, pseudo-molecular ion identified in negative ion mode.

**Table 7:** Flavonoid compounds identified in *Castanea* sp. and the standards to which they refer.

Compound	Rt (min)	${\left[M-H\right]}^{-}$ (*m*/*z*)	MS^2^ (*m*/*z*)	Expressed As
Quercetin-3-O-xyloside	19.34	433	301 (100), 300 (58)	Quercetin-3-O-glucoside
Quercetin-3-O-rutinoside	20.25	609	301 (100), 300 (27), 179 (2)	Quercetin-3-O-rutinoside
Quercetin-3-O-galactoside	21.04	463	301 (100), 300 (61), 179 (2)	Quercetin-3-O-galactoside
Quercetin-3-O-glucoside	21.22	463	301 (100), 300 (61), 179 (2)	Quercetin-3-O-glucoside
Isorhamnetin 3-O-rutinoside	21.96	623	315 (100), 300 (18)	Isorhamnetin-3-O-rutinoside
Quercetin-3-O-glucuronide	22.64	477	301(100)	Quercetin-3-O-glucuronide
Quercetin-3-O-rhamnoside	22.94	447	301 (100), 300 (26)	Quercetin-3-O-rhamnoside
Isorhamnetin 3-glucuronide	23.93	491	315 (100)	Isorhamnetin 3-rutinoside
Isorhamnetin 3-O-glucoside	24.73	477	315 (100), 433 (26)	Isorhamnetin 3-rutinoside

First or bold number, fragments that were further fragmented; Rt, retention time; [M − H]^−^, pseudo-molecular ion identified in negative ion mode; (), relative abundance of fragment ions; MS^2^, fragment ions obtained from pseudo-molecular ion in negative ion mode.

## Data Availability

The remaining data presented in this study are available on request from the corresponding author. The remaining data are not publicly available due to privacy.

## Supplementary Materials

The following supporting information can be downloaded at: https://www.mdpi.com/article/10.3390/plants13152088/s1. Table S1. Correlation coefficients between IAA in stem base (day 0) and phenolic compounds in stem cuttings (day120). Table S2. Correlation coefficients between IAA in stem base (day 0) and strigolactones in roots and callus (day 120). Table S3. Correlation coefficients between IAA in stem base (day 0) and root development (day 120). Table S4. Correlation coefficients between strigolactones concentration in roots (day 120) and root development (day 120). Table S5. Correlation coefficients between strigolactones concentration in callus (day 120) and root development (day 120). Figure S1. Pecentage of strigolactone compounds in (a) roots and (b) callus of ‘Marsol’, ‘Maraval’ and ‘Kozjak’ (day 120). Figure S2. Chromatogram of a *Castanea* sp. recorded at 350 nm.
