# Supplementary material for: Involvement of Auxin, Flavonoids and Strigolactones in the Different Rooting Ability of European Chestnut (Castanea sativa) and Hybrids (Castanea crenata × Castanea sativa)
Source: Plants (Basel). 2024 Jul 27;13(15):2088. doi: 10.3390/plants13152088 (PMC11314612; doi:10.3390/plants13152088)

Supplementary material

Table S1. Correlation coefficients between IAA in stem base (day 0) and phenolic compounds in stem cuttings (day120)

	Q -3-O-xyl	*Q -3-O-rut*	*Q -3-O-glu*	*Q- 3-O-gal*	*Iso-3-O-rut*	*Q- 3-O-glucuronide*	*Q- 3-rha*	*Iso-3-O-glucuronide*	*Iso-3-O-glu*	*TFC*
IAA	0,29	0,49	0,60	0,46	-0,05	0,47	0,59	0,52	0,53	0,65

-1 0 1

The scale of Pearson's Correlation Coefficient: 0-0.19: very low correlation; 0.2-0.39: low correlation; 0.4-0.59: moderate correlation; 0.6-0.79: high correlation; 0.8-1: very high correlation Q--3-O-xyl: Quercetin -3-O-xyloside; Q -3-O-rut: Quercetin -3-O-rutinoside; Q-3-O-glu: Quercetin -3-glucoside; Q-3-O-gal: Quercetin-3- O-galactoside; Iso-3-O-rut: Isorhamnetin-3-O-rutinoside; Q-3-O-glucuronide: Quercetin-3-O-glucuronide; Q-3-O-rha: Quercetin-3-O-rhamnoside; Iso-3-O-glucuronide: Isorhamnetin-3-O-glucuronide; Iso-3-O-glucoside: Isorhamnetin 3-O-glucoside, TPC: total flavonoid concentration. (Correlation matrix, n=15)

Table S2. Correlation coefficients between IAA in stem base (day 0) and strigolactones in roots and callus (day 120)

		Strigol	*Orobanchol*	*5-deoxystrigol*
IAA	Callus	-0,05	-0,32	0,58
IAA	Roots	0,12	0,59	0,30

-1 0 1

The scale of Pearson's Correlation Coefficient: 0-0.19: very low correlation; 0.2-0.39: low correlation; 0.4-0.59: moderate correlation; 0.6-0.79: high correlation; 0.8-1: very high correlation . (Correlation matrix, n=15)

Table S3. Correlation coefficients between IAA in stem base (day 0) and root development (day 120)

	Number of newly formed shoots	*Length of newly formed shoots*	*Number of main roots*	*Root system length*	*Callus formation*	*Acrobasal rooting*	*Basal rooting*	*Successfully rooted*
IAA	0,40	0,60	0,55	0,17	-0,37	0,47	0,03	0,50

-1 0 1

The scale of Pearson's Correlation Coefficient: 0-0.19: very low correlation; 0.2-0.39: low correlation; 0.4-0.59: moderate correlation; 0.6-0.79: high correlation; 0.8-1: very high correlation. (Correlation matrix, n=15)

Table S4. Correlation coefficients between strigolactones concentration in roots (day 120) and root development (day 120)

	Strigol	Orobanchol	5-deoxystrigol
Number of newly formed shoots	-0,07	0,07	0,14
Length of newly formed shoots	-0,58	-0,53	0,17
Number of main roots	-0,56	-0,54	0,37
Root system length	-0,46	-0,57	0,38
Callus formation	0,50	0,38	-0,31
Acrobasal rooting	-0,38	-0,29	0,20
Basal rooting	-0,02	-0,33	0,26
Successfully rooted cuttings	-0,39	-0,59	0,44

-1
0
1

The scale of Pearson's Correlation Coefficient: 0-0.19: very low correlation; 0.2-0.39: low correlation; 0.4-0.59: moderate correlation; 0.6-0.79: high correlation; 0.8-1: very high correlation . (Correlation matrix, n=15)

Table S5. Correlation coefficients between strigolactones concentration in callus (day 120) and root development (day 120)

	Strigol	Orobanchol	5-deoxystrigol
Number of newly formed shoots	0,41	0,33	0,01
Length of newly formed shoots	0,46	0,26	0,07
Number of main roots	0,34	0,20	0,12
Root system length	0,41	0,31	0,16
Callus formation	-0,33	0,03	-0,22
Acrobasal rooting	0,08	-0,10	0,21
Basal rooting	0,28	0,25	-0,13
Successfully rooted cuttings	0,32	0,14	0,07

-1
0
1

The scale of Pearson's Correlation Coefficient: 0-0.19: very low correlation; 0.2-0.39: low correlation; 0.4-0.59: moderate correlation; 0.6-0.79: high correlation; 0.8-1: very high correlation . (Correlation matrix, n=15)

Figure S1. Pecentage of strigolactone compounds in a) roots and b) callus of 'Marsol', 'Maraval' and 'Kozjak' (day 120).

(a)

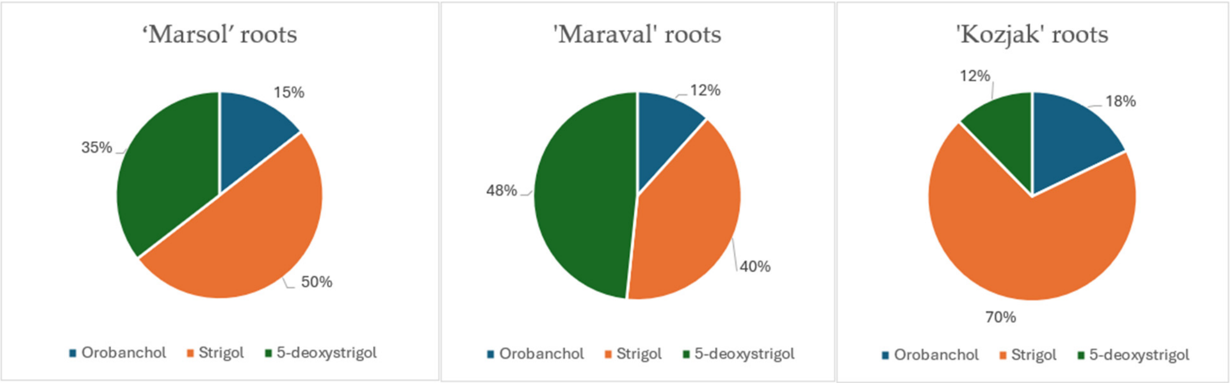

b)

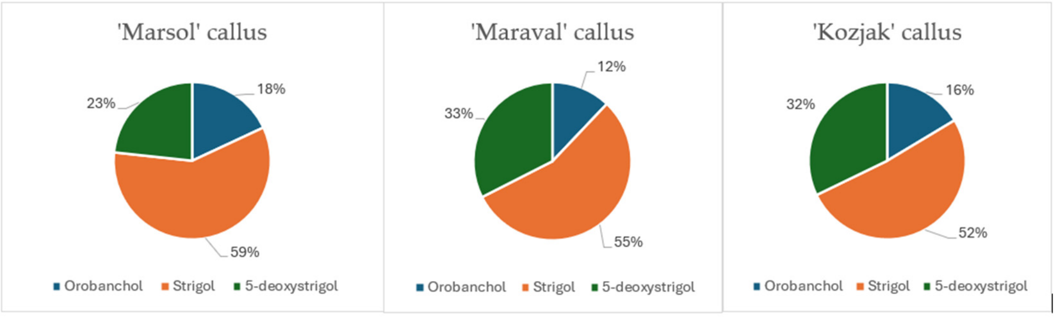

Figure S2. Chromatogram of a *Castanea* Sp. recorded at 350 nm.

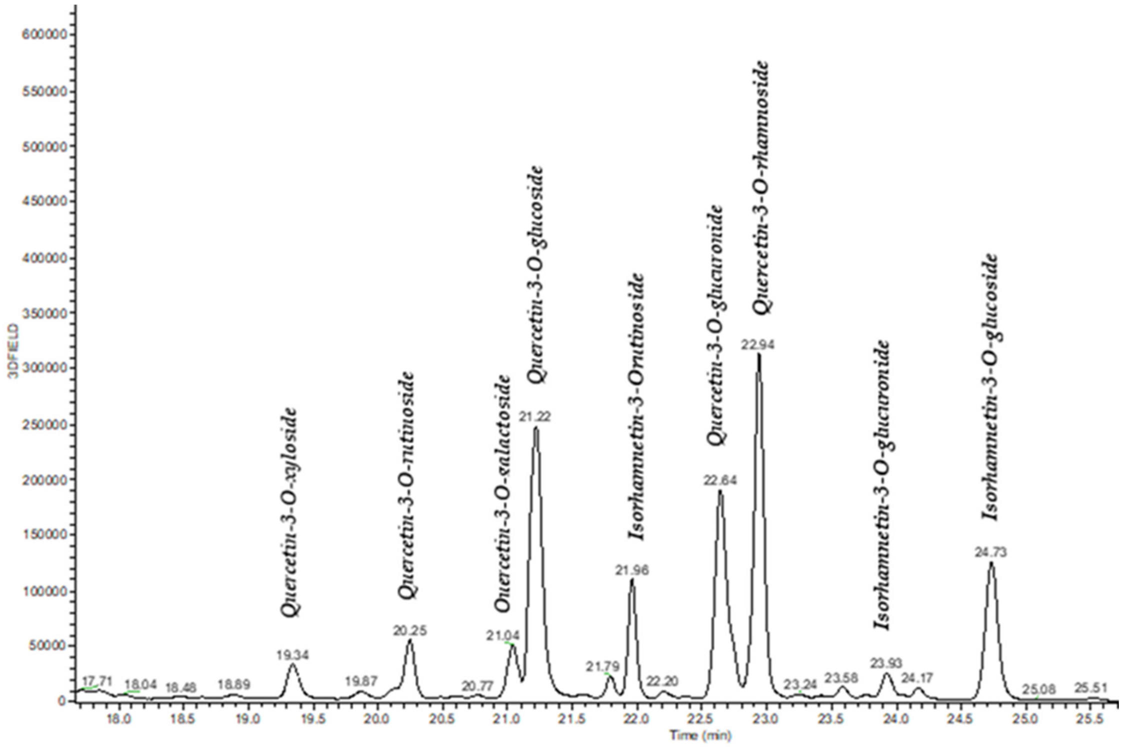

Supplement: Supplementary file 1 [file plants-13-02088-s001.zip › plants-3077372-supplementary.pdf]
